# Uterine Preservation Treatments in Sarcomas: Oncological Problems and Reproductive Results: A Systematic Review

**DOI:** 10.3390/cancers13225808

**Published:** 2021-11-19

**Authors:** Giulia Dondi, Eleonora Porcu, Alessandra De Palma, Giuseppe Damiano, Eugenia De Crescenzo, Linda Cipriani, Maria Dirodi, Gloria Ravegnini, Antonio De Leo, Margherita Nannini, Martina Ferioli, Alessio G. Morganti, Maria Abbondanza Pantaleo, Pierandrea De Iaco, Anna Myriam Perrone

**Affiliations:** 1 Division of Oncologic Gynecology, IRCCS Azienda Ospedaliero—Universitaria di Bologna, 40138 Bologna, Italy; giulia.dondi@aosp.bo.it (G.D.); eugenia.decrescenzo2@unibo.it (E.D.C.); pierandrea.deiaco@unibo.it (P.D.I.); 2 Centro di Studio e Ricerca delle Neoplasie Ginecologiche (CSR), University of Bologna, 40138 Bologna, Italy; 3 Infertility and IVF Unit, IRCCS Azienda Ospedaliero—Universitaria di Bologna, 40138 Bologna, Italy; eleonora.porcu@unibo.it (E.P.); giuseppe.damiano@aosp.bo.it (G.D.); linda.cipriani@aosp.bo.it (L.C.); maria.dirodi@aosp.bo.it (M.D.); 4 Forensic Medicine and Integrated Risk Management Unit, Azienda Ospedaliero—Universitaria di Bologna, via Albertoni 15 alessandra 40138 Bologna, Italy; alessandra.depalma@aosp.bo.it; 5 Department of Pharmacy and Biotechnology, University of Bologna, 40138 Bologna, Italy; gloria.ravegnini2@unibo.it; 6 Department of Experimental, Diagnostic and Specialty Medicine—DIMES, Alma Mater Studiorum—University of Bologna, 40138 Bologna, Italy; antonio.deleo@unibo.it (A.D.L.); alessio.morganti2@unibo.it (A.G.M.); 7 Molecular Pathology Laboratory, IRCCS Azienda Ospedaliero—Universitaria di Bologna/Azienda USL di Bologna, 40138 Bologna, Italy; 8 Division of Oncology, IRCCS Azienda Ospedaliero—Universitaria di Bologna, 40138 Bologna, Italy; margherita.nannini@unibo.it (M.N.); maria.pantaleo@unibo.it (M.A.P.); 9 Radiation Oncology, IRCCS Azienda Ospedaliero—Universitaria di Bologna, 40138 Bologna, Italy; martina.ferioli4@unibo.it

**Keywords:** fertility-sparing, gynecological cancers, sarcomas, uterine cancers, assisted reproductive treatments

## Abstract

**Simple Summary:**

Uterine sarcomas can affect patients of reproductive age. In this setting, the chance of a fertility-sparing treatment would allow women to become pregnant. In the literature, only a few experiences of fertility-sparing treatment of uterine sarcomas have been reported; however, the oncological safety and reproductive outcomes remain unclear. The aim of this systematic review is to report and summarize all the published evidence about the fertility-sparing approach in these rare and heterogenous tumors, and to help physicians in making clinical decisions.

**Abstract:**

Uterine sarcomas are rare cancers, sometimes diagnosed in women of childbearing age. Hysterectomy is the standard treatment in early stages. The option of lesion removal to save fertility is described in the literature, but it is still considered experimental. The objective of this systematic review is to report on the available evidence on the reproductive and oncological outcomes of fertility-sparing treatment in women with uterine sarcomas. PubMed, Scopus and Cochrane Central Register of Controlled Trials were searched between 1 January 2011 and 21 June 2021 for publications in English about women with uterine sarcoma treated with a fertility-sparing intervention. Thirty-seven studies were included for a total of 210 patients: 63 low-grade endometrial stromal sarcomas, 35 embryonal rhabdomyosarcomas of the cervix, 19 adenosarcomas, 7 leiomyosarcomas and 2 uterine tumors resembling an ovarian sex cord. Conservative treatment ensured pregnancy in 32% of cases. In terms of oncological outcomes, relapse was related to histology and the worst prognosis was reported for leiomyosarcoma, followed by low-grade endometrial stromal sarcoma, which relapsed in 71% and 54% of cases, respectively. The highest death rate was associated with leiomyosarcoma (57.1%). This study demonstrated that fertility-sparing treatments may be employed in selected cases of early stage uterine sarcoma.

## 1. Introduction

Uterine sarcomas represent an extremely malignant and rare group of heterogeneous tumors which grow from the uterine body and account for about 3% of all cancers in this organ [1]. They originate from the myometrium or connective tissue of the endometrium and are classified according to the criteria of the World Health Organization (WHO) [2]. Most of them have an aggressive behavior and a poor prognosis. The rarity and histopathologic heterogeneity of uterine sarcomas make the classification of risk factors, the definition of prognosis and the best treatment strategy very difficult. These are conditions that occur more frequently during menopause (60%), even though a minority of them are women of childbearing age [3].

Hysterectomy, with or without bilateral salpingo-oophorectomy, is the standard treatment in stage I disease [4]. Adnexa are systematically removed in postmenopausal women, whereas it is recommended to remove them before menopause in case of sarcoma histotypes more susceptible to hormones, such as low-grade stromal sarcomas and adenosarcomas. Leiomyosarcoma (LMS) represent the more aggressive among the group, and despite radical surgery, the risk of recurrence remains high, ranging between 50% and 70%. The high recurrence rate of LMS provides the rationale for postoperative treatment; neither adjuvant cytotoxic chemotherapy nor adjuvant radiotherapy have been shown to reduce the risk of relapse in early stages [5].

Fertility-sparing treatment (FST) is the conservative management of the reproductive system for gynecological cancers in women of childbearing age who have not yet accomplished their reproductive desires and wish to conceive. Treatment consists of removing the neoplastic lesions while preserving the uterus and at least one ovary during surgery. In recent years, many gynecological cancers, such as endometrial, cervical and even ovarian cancers diagnosed at an early stage, have been treated with a fertility-saving approach with good oncological and reproductive results [6]. For cancer patients, assisted reproductive technology (ART) techniques play an important role in fertility preservation and the achievement of pregnancy. However, some concerns about the oncologic safety of hormonal stimulation, often linked to false myths, can lead to avoiding this method in patients with hormone-sensitive tumors. For example, ART is useful for patients who wish to delay pregnancy until the end of cancer therapy by the cryopreservation of oocytes, or for women suffering from infertility because of pre-existing conditions to an anticancer treatment.

FST is considered a rare and experimental approach for uterine sarcoma because of the rarity of the event. In addition, the lack of specific guidelines for clinicians to select the most appropriate treatment and make the right counselling for a childbearing-age woman diagnosed with uterine sarcoma represents an important issue.

The aim of this systematic review is to collect and analyze all the latest experiences reported in the literature describing the FST of uterine sarcomas, in order to help physicians in counselling and making clinical decisions.

## 2. Materials and Methods

This systematic review was conducted according to the Preferred Reporting Items for Systematic Reviews and Meta-Analyses (PRISMA-P) statement [7] and was registered in Prospero on 31 July 2021 (registration number CRD42021262164). The research question was “What are reproductive and oncologic outcomes of fertility-sparing treatment in women with uterine sarcoma?”, and it was determined using the PICOS process (population, intervention, comparison, outcomes, study design) [8]. The included population was composed of women with a histological diagnosis of uterine sarcoma who wanted to preserve the reproductive function and were treated with a fertility-sparing intervention, in which the uterus and at least one ovary and fallopian tube were preserved. In this setting a comparison group was not expected. The oncologic outcomes considered were the cancer recurrence and the cancer death rate, whereas the reproductive outcomes were the total number of pregnancies, the live birth rate, the rate of assisted reproductive technology pregnancies, the rate of preterm delivery and the route of delivery. We included all types of peer-reviewed studies: randomized controlled trials (RCTs), observational, prospective and retrospective studies, case series and case reports. The review was limited to articles published between 1 January 2011 and 21 June 2021 in the English language. Studies concerning uterine tumors other than sarcomas, uterine metastases of other neoplasms, uterine sarcomas treated without preservation of the reproductive function and extra-uterine sarcomas were excluded. Moreover, we excluded abstracts, editorials, letters, comments to Editors, systematic and narrative reviews, meta-analyses without any new patient data and book chapters.

The search was performed on 1 August 2021 and conducted on PubMed, Scopus and Cochrane Central Register of Controlled Trials. The combination of free-vocabulary and/or Medical Subject Headings (MeSH) terms for the identification of studies is reported in the Supplementary Materials (Table S1). Title and abstract screening of articles was conducted independently by two authors (G.D. and E.D.C.). Duplicates were removed using a public reference manager. Studies that did not meet inclusion criteria were excluded. Full-text articles were examined independently by two reviewers (G.D. and E.D.C.). Disagreements were resolved by discussion with a third reviewer (A.M.P.). Results of the review were discussed with all authors for multidisciplinary topics. Afterwards, data from each eligible study were extracted and tabulated. The methodological quality of cohort studies was evaluated using the “Quality Assessment Tools of the National Heart, Lung, and Blood Institute” (NHLBI) [9], and for case series and case reports, by using the “Methodological quality and synthesis of case series and case reports” [10]. Institutional review board approval was not required for this study.

## 3. Results

### 3.1. Quality Assessment of Papers Included

**Figure 1:**
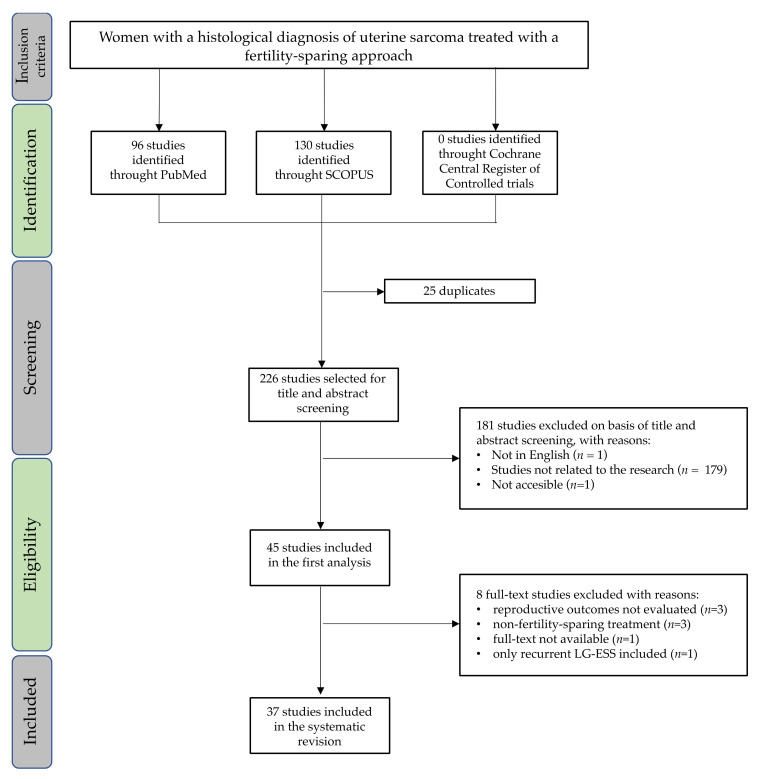
PRISMA flow chart for the studies included in the systematic review. LG-ESS: low-grade endometrial stromal sarcoma.

The initial search from electronic databases revealed 251 studies: 130 were identified using Scopus, 96 using PubMed and none using the Cochrane Central Register of Controlled Trials. A total of 25 duplicates were removed, and 226 abstracts were screened. There were 45 full-text articles assessed for eligibility; only 37 studies were included in the final analysis, as shown in Figure 1. Of those, 18 were case reports, 4 were case series, 14 were retrospective studies and 1 was a prospective study. The studies included in this review were all published between 1 January 2011 and 21 June 2021.

The risk of bias is reported in Table 1 and Table 2. The quality of 14 retrospective studies and of the prospective study was rated as “fair” in all cases. The most common biases were the absence of sample size justification, the lack of blinded assessors to the exposure of participants and the missed measurement of confounding variables. The quality for 4 case series and 18 case reports was rated as “good” in 17 studies, and “fair” in 5 studies, as observed in Table 2. Five studies were rated as “fair” quality due to the length of follow up not being long enough for outcomes to occur.

### 3.2. Characteristics of Patients Included

Table 3, Table 4, Table 5, Table 6 and Table 7 summarize data from a total of 210 patients treated with FST following the diagnosis of uterine sarcoma. Findings were stratified by histotypes and included 63 (30%) low-grade endometrial stromal sarcomas (LG-ESS), 35 (17%) embryonal rhabdomyosarcomas (RMS) of the cervix, 19 (9%) adenosarcomas, 7 (3%) LMS and 2 (1%) uterine tumors resembling ovarian sex cord tumors (UTROSCT). Patients with a histologic diagnosis of smooth muscle tumor of uncertain malignant potential (STUMP) were also included in our analysis and represent the largest group of patients (*n* = 84, 40%).

The data indicated that patients requiring FST were nulliparous in most cases (166, 79%), but can also have had pregnancies in the past (14, 7%); for three patients, information was unavailable. In an analyzed study, the data were difficult to retrieve because a median of zero pregnancies with a range of 0 to 3 was reported without other details [21].

### 3.3. Reproductive Outcomes

The reproductive outcomes assessed in this study were the overall number of pregnancies, the ART pregnancy rate, the number of spontaneous abortions, the rate of premature births and the Caesarean section (C-section) rate, as indicated in Table 8.

Among 210 patients receiving FST, 67 conceived (32%). A total of 68 pregnancies were achieved, because 1 patient with LG-ESS had 2 pregnancies because FST (tumor resection) was performed during the C-section of the first pregnancy, and they gave birth a second time after 45 months. Of the 68 pregnancies, only 17 (25%) received ART support. The live births were 64 (94%), derived from 59 patients with a term delivery (87%) and 5 with premature delivery; only 4 patients had spontaneous abortions in their first trimester. A histotype analysis reported that about half of conservatively treated patients with LG-ESS, STUMP and LMS conceived, whereas patients with adenosarcoma and embryonal RMS had a lower pregnancy rate than the others (21% and 9%, respectively). Patients with LMS and STUMP requested ART more frequently (33% and 38%, respectively) and the 2 patients with UTROSCT had both spontaneous pregnancies. C-sections were performed in 66% of cases, and the reported percentage varied widely between sarcoma histotypes and the type of FST performed: patients with LMS/STUMP and LG-ESS reported the highest percentage of C-section (72% and 70%, respectively). All patients with adenosarcoma had a vaginal delivery, whereas for UTROSCT and embryonal RMS of the cervix, the caesarean delivery rates were 50% and 33%, respectively.

### 3.4. Oncological Treatments and Outcomes

Different surgical approaches were adopted based on the site (uterine body or uterine cervix) and histotypes of lesions; data are summarized in Table 9.

Patients with LG-ESS had laparotomic, laparoscopic or hysteroscopic tumor resection in similar rates (21%, 21% and 18%, respectively). Adenosarcomas were treated predominantly with a hysteroscopic approach (84%). Laparotomic myomectomy was performed mostly in patients with STUMP (31%). Embryonal RMS of the cervix was treated with cervical excision, conization (68%) or, less frequently, trachelectomy (23%). About half of patients included in this analysis (44%) received a conservative treatment (tumor resection) without any notice about the surgical approach. The type of adjuvant therapy used after fertility-sparing surgery is reported and summarized in Table 10.

Neoadjuvant chemotherapy (NACT) was used in 5 patients with embryonal RMS of the cervix; different chemotherapy regimens were described: combinations of vincristine, ifosfamide, dactinomycin, cyclophosphamide and irinotecan (Table 5). Adjuvant treatment was used in 71 (34%) patients: 40 (56%) of them had hormone therapy (LG-ESS and adenosarcoma) and the most common drugs used were megestrol acetate, medroxyprogesterone acetate or gonadotrophin-releasing hormone agonists; 31 (44%) had chemotherapy with carboplatin and paclitaxel for LG-ESS (Table 3), with ifosfamide and cisplatin for adenosarcomas (Table 4), with ifosfamide and nimustine for LMS (Table 5) and with combinations of vincristine, dactinomycin, cyclophosphamide, doxorubicin, dacarbazine, etoposide, cisplatin and bleomycin for embryonal RMS (Table 7). The majority of the patients (64%) did not receive any medical treatment after surgery. Particularly, no cases of STUMP or UTROSCT were treated with NACT or adjuvant treatments. Almost all patient candidates for FST at diagnosis had a disease confined to the uterus (stage I). The oncologic outcomes considered have been cancer recurrence rate, cancer death rate and number of recurrences during pregnancy (Table 11).

In total, 56 out of 210 patients had a recurrence (27%). The highest recurrence rate was recorded in patients with LMS (*n* = 5, 71%), followed by LG-ESS (*n* = 34, 54%). The number of recurrences was lower in women treated for adenosarcoma (*n* = 3, 16%), STUMP (*n* = 9, 11%) and embryonal RMS of the cervix (*n* = 5, 14%). Recurrence during pregnancy, which is a rare event, was experienced by three patients with LG-ESS: one with LMS and one with embryonal RMS of the cervix. Preterm delivery was associated with recurrence during pregnancy in those women.

Considering all histotypes together, the death rate was 3%; the highest percentage was observed in LMS, with the death of four patients (57%) after disease recurrence. One death was reported among patients with LG-ESS and with STUMP, and in the last case, the recurrence was as LMS.

## 4. Discussion

In our systematic review, we have summarized and analyzed the current available literature on FST in uterine sarcomas. In addition, we have performed a critical assessment of the quality of studies included, presenting an overview of the fertility-saving options in different types of uterine sarcomas. The review covered two areas: oncologic and reproductive outcomes. Most of the data extracted came from historical cohort studies and case series describing a heterogeneous population where information on relapses and deaths was frequently lacking, making comparisons between studies and patient’s groups impossible.

However, with those limitations in mind, we can learn significant lessons from this analysis, although judgement is limited by the small number of cases (only 210 patients). First, FST could be a possible and well described approach in uterine sarcomas; the sparing of the uterus by enucleation of the lesion can be carried out by MIS (hysteroscopy, laparoscopy) or open access; moreover, none of these approaches was identified as the preferred. In general, the MIS approach was reported as a preferred method for reproductive problems [48], but it was not considered the safest approach in oncology. The question of what the best surgical approach is remains unsolved because the available data do not allow for a comparison between oncology and reproductive outcomes of MIS and open surgery. The literature data showed an increased risk for relapse and death related to the surgical tumor fragmentation. It is important to stress that this practice should be avoided in FST; however, we are aware that some cases of hysteroscopic morcellation are hard to avoid, especially if the purpose is saving the uterus. Sarcomas are a very heterogeneous class of diseases and can therefore be analyzed by histotype, stage, grade and growth profile; the results of FST and standard treatments could be considered, although this type of analysis requires a large series of patients, which is currently not available. Moreover, the role of postoperative treatment in uterine sarcomas is still debated, and current decision-making varies by histological subtype and other pathological and clinical features, in a case-by-case fashion [5]. Finally, the choice of a conservative surgery should take into account the biological background of uterine sarcomas, considering that the pathogenetic role of estrogen varies among different histotypes.

Given these considerations, we observed that, in certain types of sarcomas, conservative treatment may be an acceptable option, whereas for other types, due to poor survival, the treatment might be deterred by waiting for more data.

An example of survival aggravated by FST was reported in the LMS group, where four out of seven patients died of disease; the FST group showed an OS of 43%, which is much less than the standard treatment (73% based on data published in the literature) [49]. Another interesting group was the LG-ESS, where the recurrence rate in the FST appeared to be higher (54%) compared to the standard treatment, although this increase in recurrence did not lead to a higher risk of death (2%). Typically, this type of sarcoma shows delayed relapses (around 60% of cases), and a slow progression of the disease.

Our review showed that patients with adenosarcoma did not report relapse after FST. In general, adenosarcoma without overgrowth features has a good prognosis, and FST does not seem to worsen it. Among STUMPs managed with standard treatments, the risk of recurrence ranges between 0% and 28% and depends on cytologic atypia, mitotic index and the necrosis of tumor cells. In our review, STUMP patients subjected to FST displayed a relapse of 10%. Thus, patients with STUMP are possible candidates for FST, although STUMPs could naturally recur as LMS. Accordingly, in our series, we reported that one death occurred in the STUMP group due to these reasons. Finally, because of the limited number of reported cases, no indication can be given for UTROSCT and RMS; however, our analysis indicates that there were no adverse results.

When looking to the impact of uterine savings on fertility, we observed that these treatments led to pregnancy in 32% of cases (67 patients conceived), and adenosarcomas had the lowest pregnant rate (21%). Assumptions may be different: related to the site of origin of the cancers (within the uterine cavity) or to the absence of ART programs, for the risk of stimulating a potentially hormone-sensitive tumor.

However, analyzing the use of ART, only 17 patients in our analysis had used ART after FST; the diagnosis was LG-ESS, STUMP and LMS. These data raise the question of whether ART can be considered an integral part of uterine-sparing treatment. A second hypothesis could be that many patients undergoing FST are not interested in becoming pregnant after the treatment. Therefore, these data on post-treatment fertility rates may be influenced by many unknown factors not published in the literature.

Considering the delivery methods, based on our series, 66% of patients underwent C-sections; this percentage reached 100% for LMS, about 70% for LG-ESS and STUMP, and 0% for adenosarcomas. There are several factors involved in this decision as well. The choice of C-section versus natural delivery could be related to the type of FST; for example, adenosarcomas were almost all excised with operative hysteroscopy, whereas RMS involved the cervix and almost all underwent minor procedures (loop electrosurgical excision procedure, local excision), allowing a spontaneous delivery in both situations. The other cancers involved myometrium, and the common treatments reported were hysterotomies with open surgery or minimally invasive surgery (MIS). However, despite the increase in cesarean section use, FST has not resulted in an increase in obstetric risk.

What should women who have conceived after FST do? Is there any indication to radicalize surgery with a hysterectomy? Analysis of these cases shows a lack of indications post-pregnancy. Hysterectomy could potentially protect against uterine recurrence, especially in low-aggressive tumors such as LG-ESS and adenosarcomas, but the impact on OS of this practice is also unknown.

It is important to point out that if saving the uterus is not possible, in some countries, surrogacy can be an alternative for women who wish to have babies, but the impact of this procedure has not yet been evaluated in women with gynecological cancers [50].

This study has many strengths: (i) it is the first systematic review on this underreported topic; (ii) it provides a complete overview of the fertility-sparing treatment in different histotypes of uterine sarcoma; and (iii) it reports both oncologic and reproductive outcomes.

The main limitations of this revision are: (i) it was derived from very small series or case reports, including only one prospective study on FST in LG-ESS analyzing six patients [13]; (ii) the lack of a long-term follow-up to better define the risk of relapse and death; (iii) the absence of a clearly defined pre-treatment fertility assessment based on specific guidelines for ART and ART clinicians; (iv) a publication bias may have been present because authors tend to avoid publishing negative results; however, previous reports on fertility preservation in cancer patients undergoing cryopreservation of ovarian tissues have also shown that only a minority of women would use the option of obtaining pregnancy; and (v) a lack of safety data for MIS and hysteroscopic fragmentation.

## 5. Conclusions

FST is a possible option foreseen in gynecological cancers, used in cervical, endometrial, and ovarian cancer at stage I, which produces acceptable oncological and reproductive outcomes. The purpose of this review was to investigate whether FST can also be considered for uterine sarcomas. The data do not allow us to make a definitive judgment, but our results may suggest that, except for LMS, FST could be a reasonable treatment option for young women who wish to have children. However, close surveillance and long-term follow-up and the possibility of post-pregnancy radicalization should be mandatory in these cases. Given the complexity and risk of recurrence and death of these tumors, expert advice is needed to help patients make the decision of whether to undergo FST; however, it is difficult to become an expert due to the rarity of the situation. Therefore, fertility-saving decisions are not only challenging for the patient, but also for the treating oncologist–gynecologist team. Consequently, the management of these patients should involve multidisciplinary and experienced teams in tertiary referral centers with large populations with uterine sarcoma. Furthermore, participation in clinical trials is essential because it is the only way to evaluate and improve the quality of care. International cooperation would help to increase patient numbers and develop guidelines.

## Figures and Tables

**Table 1:** Quality assessment tool for the observational cohort and cross-sectional studies.

Study, Year [Ref]	Criteria													
	1	2	3	4	5	6	7	8	9	10	11	12	13	14
LG-ESS														
Tunc et al. 2019 [11]							NA	NA		NA		NA		
Xie et al., 2017 [12]							NA	NA		NA		NA		
Laurelli et al., 2015 [13]							NA	NA		NA		NA		
Jin et al., 2015 [14]							NA	NA		NA		NA		
Bai et al., 2014 [15]							NA	NA		NA		NA		
Adenosarcoma														
Yuan et al. 2019 [16]							NA	NA		NA		NA		
Togami et al. 2018 [17]							NA	NA		NA		NA		
Lee et al. 2017 [18]							NA	NA		NA		NA		
STUMP														
Ning et al. 2021 [19]							NA	NA		NA		NA		
Shim et al. 2020 [20]							NA	NA		NA		NA		
Şahin at al., 2019 [21]							NA	NA		NA		NA		
Ha et al., 2018 [22]							NA	NA		NA		NA		
Embryonal RMS of the uterine cervix														
Ricciardi et al., 2020 [23]							NA	NA		NA		NA		
Dehner et al., 2012 [24]							NA	NA		NA		NA		
Li et al., 2011 [25]							NA	NA		NA		NA		

1. Was the research question or objective in this paper clearly stated? 2. Was the study population clearly specified and defined? 3. Was the participation rate of eligible persons at least 50%? 4. Were all the subjects selected or recruited from the same or similar populations (including the same time period)? Were inclusion and exclusion criteria for being in the study prespecified and applied uniformly to all participants? 5. Were a sample size justification, power description, or variance and effect estimates provided? 6. For the analyses in this paper, were the exposure(s) of interest measured prior to the outcome(s) being measured? 7. Was the timeframe sufficient so that one could reasonably expect to observe an association between exposure and outcome if it existed? 8. For exposures that can vary in amount or level, did the study examine different levels of the exposure as related to the outcome (e.g., categories of exposure, or exposure measured as continuous variable)? 9. Were the exposure measures (independent variables) clearly defined, valid, reliable, and implemented consistently across all study participants? 10. Was the exposure(s) assessed more than once over time? 11. Were the outcome measures (dependent variables) clearly defined, valid, reliable and implemented consistently across all study participants? 12. Were the outcome assessors blinded to the exposure status of participants? 13. Was loss to follow-up after baseline 20% or less? 14. Were key potential confounding variables measured and adjusted statistically for their impact on the relationship between exposure(s) and outcome(s)? Legend: 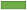: yes; 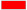: no; LG-ESS: low-grade endometrial stromal sarcoma; NA: not applicable; RMS: rhabdomyosarcoma; STUMP: smooth muscle tumor of uncertain malignant potential.

**Table 2:** Quality assessment of case series and case reports.

Study, Year [Ref]	Selection	Ascertainment		Causality				Reporting
	1	2	3	4	5	6	7	8
LG-ESS								
Gu et al. 2021 [26]					NA	NA		
Seong et al. 2020 [27]					NA	NA		
Chin et al. 2018 [28]					NA	NA		
Morimoto et al. 2015 [29]					NA	NA		
Noventa et al. 2015 [30]					NA	NA		
Dong et al. 2014 [31]					NA	NA		
Dong et al. 2014 [32]					NA	NA		
Choi et al. 2014 [33]					NA	NA		
Sánchez-Ferrer et al. 2013 [34]					NA	NA		
Delaney et al. 2012 [35]					NA	NA		
Adenosarcoma								
Zizolfi et al. 2021 [36]					NA	NA		
Goh et al. 2018 [37]					NA	NA		
UTROSCT								
Carbone et al. 2021 [38]					NA	NA		
Embryonal RMS of the uterine cervix								
Bell et al. 2021 [39]					NA	NA		
Moufarrij et al. 2020 [40]					NA	NA		
Buruiana et al. 2020 [41]					NA	NA		
John et al. 2018 [42]					NA	NA		
May et al. 2018 [43]					NA	NA		
Bouchard-Fortier et al. 2016 [44]					NA	NA		
Ayas et al. 2015 [45]					NA	NA		
Strahl et al. 2012 [46]					NA	NA		
Sobiczewski et al. 2011 [47]					NA	NA		

1. Does the patient(s) represent(s) the whole experience of the investigator (center) or is the selection method unclear to the extent that other patients with similar presentation may not have been reported? 2. Was the exposure adequately ascertained? 3. Was the outcome adequately ascertained? 4. Were other alternative causes that may explain the observation ruled out? 5. Was there a challenge/rechallenge phenomenon? 6. Was there a dose–response effect? 7. Was follow-up long enough for outcomes to occur? 8. Is the case(s) described with sufficient details? Legend: 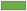: yes; 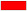: no; LG-ESS: low-grade endometrial stromal sarcoma; NA: not applicable; RMS: rhabdomyosarcoma; STUMP: smooth muscle tumor of uncertain malignant potential; UTROSCT: uterine tumor resembling ovarian sex cord tumor.

**Table 3:** Published reproductive and oncologic outcomes after FST of LG-ESS.

Study, Year [Ref]	Design	FST Patients (*n*)	Age (Median, Range)	Parity	Surgery	Adjuvant Treatment	Follow Up (Median, Range)	Recurrence, *n* (%)	Death, *n* (%)	Total Pregnancies (*n*)	ART (*n*)	Details
Gu, 2021 [26]	Case report	1	28	NU	Laparotomic myomectomy during C-section	-	96	0	0	2	0	Full-term C-section: 2
Seong, 2020 [27]	Case report	1	24	NU	Laparoscopic myomectomy	MA 320 mg/day	35	0	0	0	-	-
Tunc, 2019 [11]	Retrospective	13 (LG-ESS: 6; LMS: 7)	31.5 (26–35)	PS: 1, NU: 5	Excision of mass: 5 Excision of mass + staging surgery: 1	MA: 2 CBDCA + PTX: 1 no: 3	61.5 (13–142)	4 (66.7)	0	1	1	No live birth
Chin, 2018 [28]	Case report	1	34	NU	Laparotomic myomectomy	MA 160 mg/day followed by 80 mg/day	84	100	0	0	-	-
Xie, 2017 [12]	Retrospective	17	28 (15–37)	NU: 15; PS: 2	Myomectomy (laparotomic: 5; laparoscopic: 7; hysteroscopic: 5)	MPA or MA: 9; GnRHa: 4; GnRHa + LNG-IUS: 2	39 (4–106)	10 (58.8)	0	5	1	Full-term C-section: 4 Pre-term C-section (29 W): 1 2 Recurrences during pregnancy
Laurelli, 2015 [13]	Prospective	6	33.5 (18–40)	NU: 4; PS: 2	HR: 6	MA 160 mg/day: 5	43.5 (30–70)	0	0	3	0	First trimester miscarriage: 1 Full-term VB: 2
Jin, 2015 [14]	Retrospective	5	32 (28–37)	NU:5	Myomectomy (laparotomic: 1; laparoscopic: 4)	MA 4; GnRHa 1	33 (10–39)	2 (40)	0	3	1	Full-term C-section: 3
Morimoto, 2015 [29]	Case report	1	25	NU	Laparotomic resection	MPA 600 mg/die	120	1 (100)	1 (100)	0	-	-
Noventa, 2015 [30]	Case report	1	34	NU	Laparoscopic myomectomy	-	15	0	0	1	0	Ongoing
Dong, 2014 [31]	Case report	1	19	NU	Laparotomic myomectomy, resection of intestinal nodule, partial omentectomy	MA 250 mg/day	33	0	0	0	-	-
Bai, 2014 [15]	Retrospective	19	NA	NA	Myomectomy: 19	NA	20.5 (3–53)	15 (78.9)	1 (5.3)	8	0	Full-term C-section: 5 Full-term VB: 3
Dong, 2014 [32]	Case report	1	25	NU	Laparotomic myomectomy	MPA 250 mg/day	32	0	0	1	0	Full-term C-section
Choi, 2014 [33]	Case report	1	31	NU	HR + PLND + photodynamic therapy	Letrozole 2.5 mg	99	0	0	1	0	Twin pregnancy. Preterm C-section (32 W)
Sánchez-Ferrer, 2013 [34]	Case report	1	32	NU	Laparotomic myomectomy	MA 80 mg/day	60	1 (100)	0	1	1	Twin dichorionic–diamniotic pregnancy. P-prom and C-section at 32 W. Recurrence during pregnancy
Delaney, 2012 [35]	Case report	1	16	NU	Laparotomic myomectomy	MA	96	0	0	1	0	Preterm C-section (34 W)

ART: assisted reproductive technology; CBDCA: carboplatin; C-section: caesarean section; FST: fertility-sparing treatment; GnRHa: gonadotrophin-releasing hormone agonists; HR: hysteroscopic resection; LG-ESS: low-grade endometrial stromal sarcoma; LMS: leiomyosarcoma; LNG-IUS: levonorgestrel-releasing intrauterine system; MA: megestrol acetate; MPA: medroxyprogesterone acetate; *n*: number; NA: not available; NU: nulliparous; PLND: pelvic lymph node dissection; P-prom: preterm premature rupture of membranes; PTX: paclitaxel; VB: vaginal birth; W: weeks.

**Table 4:** Published reproductive and oncologic outcomes after FST of adenosarcoma.

Study, Year [Ref]	Design	FST Patients (*n*)	Age (Median, Range)	Parity	Surgery	Adjuvant Treatment	Follow Up (Median, Range)	Recurrence, *n* (%)	Death, *n* (%)	Total Pregnancies (*n*)	ART (*n*)	Details
Zizolfi, 2021 [36]	Case report	1	23	NU	HR	MA 160 mg/day	60	0	0	1	0	Full-term VB
Yuan, 2019 [16]	Retrospective	9 (cervical: 5; uterine body: 4	25 (19–34)	NA	HR: 9	CT: 3 MA/MPA: 3 No: 3	19 (15–62)	1 (11.1)	0	1	0	Full-term VB
Goh, 2018 [37]	Case report	1	21	NU	Polipectomy	-	139	1 (100)	0	1	-	Full-term VB
Togami, 2018 [17]	Retrospective	1 (cervical)	17	NU	Conization	-	62	0	0	0	-	-
Lee, 2017 [18]	Retrospective	7	27 (21–40)	NU: 7	HR: 5; cervical excision: 1; dilatation and curettage: 1	MPA: 1; CT (IFO + DDP): 1 No: 5	NA	2 (28.6)	0	1	0	Full-term VB

ART: assisted reproductive technology; CT: chemotherapy; DDP: cisplatin; FST: fertility-sparing treatment; HR: hysteroscopic resection; IFO: ifosfamide; MA: megestrol acetate; MPA: medroxyprogesterone acetate; *n*: number; NU: nulliparous; VB: vaginal birth.

**Table 5:** Published reproductive and oncologic outcomes after FST of STUMP and LMS.

Study, Year [Ref]	Design	FST Patients (*n*)	Age (Median, Range)	Parity	Surgery	Adjuvant Treatment	Follow Up (Median, Range)	Recurrence, *n* (%)	Death, *n* (%)	Total Pregnancies (*n*)	ART (*n*)	Details
Ning, 2021 [19]	Retrospective	2 (STUMP)	36 (34–38)	NA	Laparoscopic myomectomy: 2	-	74.5 (71–78)	0	0	2	0	Full-term C-section: 2
Shim, 2020 [20]	Retrospective	48 (STUMP)	32.5 (20–46)	NU: 44; PS: 4	Myomectomy: 48	-	28.5 (7–130)	3 (6.3)	0	16	6	Full-term C-section: 10; Ongoing: 4; First trimester miscarriage: 2
Tunc, 2019 [11]	Retrospective	13 (LG-ESS: 6; LMS: 7)	28 (23–37)	NU: 4; PS: 3	Excision of mass: 3; excision of mass + staging surgery: 4	CT (IFO + ACNU): 3 No: 4	31 (16–109)	5 (71.4)	4 (57.1)	3	1	Full-term C-section: 2; preterm C-section (28 W): 1. Recurrence during pregnancy: 1
Şahin, 2019 [21]	Retrospective	27 (STUMP)	37 (23–52)	0 (0–3)	Laparotomic myomectomy: 26; hysteroscopic myomectomy: 1	-	58 (16–125)	6 (22.2)	1 (3.7)	7	3	Full-term C-section: 5; preterm C-section (35 W): 1; full-term VB: 1
Ha, 2018 [22]	Retrospective	7 (STUMP)	32 (28–48)	NU 6; PS 1	Myomectomy: 7	-	NA	0	0	4	3	Full-term C-section: 3, ongoing: 1

ART: assisted reproductive technology; ACNU: nimustine; C-section: caesarean section; CT: chemotherapy; FST: fertility-sparing treatment; IFO: ifosfamide; LG-ESS: low-grade endometrial stromal sarcoma; LMS: leiomyosarcoma; *n*: number; NA: not available; NU: nulliparous; PS: parous; STUMP: smooth muscle tumor of uncertain malignant potential; VB: vaginal birth; W: weeks.

**Table 6:** Published reproductive and oncologic outcomes after FST of UTROSCT.

Study, Year [Ref]	Design	FST Patients (*n*)	Age (Median, Range)	Parity	Surgery	Adjuvant Treatment	Follow Up (Median, Range)	Recurrence, *n* (%)	Death, *n* (%)	Total Pregnancies (*n*)	ART (*n*)	Details
Carbone, 2021 [38]	Case series	2	27.5 (25–30)	NUs: 2	Dilatation and curretage: 1; Laparotomic myomectomy: 1	-	56 (16–96)	0	0	2	0	Full-term C-section: 1; Full-term VB: 1

ART: assisted reproductive technology; C-section: caesarean section; FST: fertility-sparing treatment; *n*: number; NU: nulliparous; UTROSCT: uterine tumor resembling ovarian sex cord tumor; VB: vaginal birth.

**Table 7:** Published reproductive and oncologic outcomes after FST of embryonal RMS of the uterine cervix.

Study, Year [Ref]	Design	FST Patients (*n*)	Age (Median, Range)	Parity	Surgery	Adjuvant Treatment	Follow Up (Median, Range)	Recurrence, *n* (%)	Death, *n* (%)	Total Pregnancies (*n*)	ART (*n*)	Details
Bell, 2021 [39]	Case series	2	16.5 (16–17)	NU: 2	Excision of cervical mass: 2	6 courses of VAC: 2	NA	0	0	0	-	-
Moufarrij, 2020 [40]	Case report	1	17	NU	Excision of cervical mass	3 courses of DOX, DTIC, CTX and VCR	324	0	0	1	-	Full-term VB
Buruiana, 2020 [41]	Case series	2	15.5 (15–16)	NU: 2	Simple trachelectomy: 2	4 courses of NACT with VCR, IFO, dactinomycin + 5 courses of adjuvant VIA: 2	42 (24–60)	0	0	0	-	-
Ricciardi, 2020 [23]	Retrospective	5	23 (20–37)	NU: 4; PS: 1	Local excision: 5	3 or 4 courses of DOX + IFO: 2; unknown CT: 1 No CT: 2	186 (23–282)	2 (40)	0	1	0	Recurrence during pregnancy
John, 2018 [42]	Case report	1	13	NU	Local excision + LEEP after CT	6 courses of VCR, DOX and CTX	24	0	0	0	-	-
May, 2018 [43]	Case report	1	2	NU	Hysteroscopic resection + Laparoscopic Radical Trachelectomy after NACT	2 courses of NACT (VAC/VCR and irinotecan) + 14 courses of adjuvant CT (ARST0531 trial protocol)	NA	0	0	0	-	-
Bouchard-Fortier, 2016 [44]	Case series	3	20 (14–21)	NU: 3	Radical trachelectomy: 2. hysteroscopic resection + cervical conization: 1	VAC alternating with VCR and irinotecan (43 W): 1; 4 courses of VAC + 4 courses of VA: 1; 6 courses of VAC (NACT): 1	10 (8–22)	0	0	0	-	-
Ayas, 2015 [45]	Case report	1	21	NU	Conizazion + laparoscopic PLND after pregnancy	3 courses of DOX + IFO and MESNA (NACT)	16	0	0	1	0	Incidental pregnancy 7 weeks after the end of NACT. Full-term C-section
Strahl, 2012 [46]	Case report	1	18	NU	Tumor resection + laparoscopic PLND	4 courses of IFO, VCR and dactinomycin	24	0	0	0	1	Cryoconservation of ovarian tissue
Dehner, 2012 [24]	Retrospective	14	13 (9–32)	NU 14	Polipectomy + LEEP: 12; biopsy: 2	CT (VAC): 13; no: 1	24 (0–216)	3	0	NA	-	-
Li, 2011 [25]	Retrospective	3	13 (11–15)	NU: 3	Abdominal radical trachelectomy: 3	4 courses of CTX + VCR + KSM: 2; 4 courses of BLM + DDP + VP16: 1	NA	0	0	0	-	-
Sobiczewski, 2011 [47]	Case report	1	22	NU	Polipectomy	6 courses of VCR and dactinomycin	71	0	0	1	0	Full-term VB

ART: assisted reproductive technology; BLM: bleomycin; C-section: caesarean section; CT: chemotherapy; CTX: cyclophosphamide DDP: cisplatin; DOX: doxorubicin; DTIC: dacarbazine; FST: fertility-sparing treatment; IFO: ifosfamide; KSM: actinomycin K; LEEP: loop electrosurgical excision procedure; m: months; *n*: number; NA: not available; NACT: neoadjuvant chemotherapy; NU: nulliparous; PLND: pelvic lymph node dissection; PS: parous; RMS: rhabdomyosarcoma; VAC: vincristine, dactinomycin and cyclophosphamide; VB: vaginal birth; VCR: vincristine; VIA: vincristine, ifosfamide and doxorubicin; VP16: etoposide; W: weeks.

**Table 8:** Reproductive outcomes after FST of uterine sarcomas.

Sarcoma	Patients, *n*	Pregnancies, *n* (%)	ART, *n* (%)	Miscarriages, *n* (%)	Preterm Deliveries, *n* (%)	C-Section, *n* (%)
LG-ESS	63	27 (43)	5 (19)	2 (7)	4 (15)	19 (70)
Adenosarcoma	19	4 (21)	0	0	0	0
STUMP	84	29 (35)	11 (38)	2 (7)	0	21 (72)
LMS	7	3 (43)	1 (33)	0	1 (33)	3 (100)
UTROSCT	2	2 (100)	0	0	0	1 (50)
Embryonal RMS of uterine cervix	35	3 (9)	0	0	0	1 (33)
Total	210	68 (32)	17 (25)	4 (6)	5 (7)	45 (66)

ART: assisted reproductive technology; C-section: caesarean section; FST: fertility-sparing treatment; LG-ESS: low-grade endometrial stromal sarcoma; LMS: leiomyosarcoma; *n*: number; RMS: rhabdomyosarcoma; STUMP: smooth muscle tumor of uncertain malignant potential; UTROSCT: uterine tumor resembling ovarian sex cord tumor.

**Table 9:** Surgical approach for FST of uterine sarcomas.

Sarcoma	Patients, *n*	Laparotomic Myomectomy, *n* (%)	Laparoscopic Myomectomy, *n* (%)	Hysteroscopic Myomectomy/Tumor resection, *n* (%)	Cervical Excision/LEEP, *n* (%)	Trachelectomy	Other/Not Specified, *n* (%)
LG-ESS	63	13 (21)	13 (21)	12 (18)	0	0	25 (40)
Adenosarcoma	19	0	0	16 (84)	2 (11)	0	1 (5)
STUMP	84	26 (31)	2 (2)	1 (1)	0	0	55 (66)
LMS	7	0	0	0	0	0	7 (100)
UTROSCT	2	1 (50)	0	0	0	0	1 (50)
Embryonal RMS of uterine cervix	35	0	0	0	24 (68)	8 (23)	3 (9)
Total	210	40 (19)	15 (7)	29 (14)	26 (12)	8 (4)	92 (44)

FST: fertility-sparing treatment; LEEP: loop electrosurgical excision procedure; LG-ESS: low-grade endometrial stromal sarcoma; LMS: leiomyosarcoma; *n*: number; RMS: rhabdomyosarcoma; STUMP: smooth muscle tumor of uncertain malignant potential; UTROSCT: uterine tumor resembling ovarian sex cord tumor.

**Table 10:** Medical therapy in FST of uterine sarcomas.

Sarcoma	Patients, *n*	Neoadjuvant CT, *n* (%)	Adjuvant CT, *n* (%)	Adjuvant Hormone Therapy, *n* (%)
LG-ESS	63	0	1 (2)	35 (56)
Adenosarcoma	19	0	4 (21)	5 (26)
STUMP	84	0	0	0
LMS	7	0	3 (43)	0
UTROSCT	2	0	0	0
Embryonal RMS of uterine cervix	35	5 (14)	23 (66)	0
Total	210	5 (2)	31 (15)	40 (19)

CT: chemotherapy; FST: fertility-sparing treatment; LG-ESS: low-grade endometrial stromal sarcoma; LMS: leiomyosarcoma; *n*: number; RMS: rhabdomyosarcoma; STUMP: smooth muscle tumor of uncertain malignant potential; UTROSCT: uterine tumor resembling ovarian sex cord tumor.

**Table 11:** Oncologic outcomes after FST of uterine sarcomas.

Sarcoma	Patients, *n*	Recurrences, *n* (%)	Recurrences during Pregnancy, *n*	Death, *n* (%)
LG-ESS	63	34 (54)	3	1 (2)
Adenosarcoma	19	3 (16)	0	0
STUMP	84	9 (11)	0	1 (1)
LMS	7	5 (71)	1	4 (57)
UTROSCT	2	0	0	0
Embryonal RMS of uterine cervix	35	5 (14)	1	0
Total	210	56 (27)	5	6 (3)

FST: fertility-sparing treatment; LG-ESS: low-grade endometrial stromal sarcoma; LMS: leiomyosarcoma; *n*: number; RMS: rhabdomyosarcoma; STUMP: smooth muscle tumor of uncertain malignant potential; UTROSCT: uterine tumor resembling ovarian sex cord tumor.

## Data Availability

No new data were created or analyzed in this study. Data sharing is not applicable to this article.

## Supplementary Materials

The following are available online at https://www.mdpi.com/article/10.3390/cancers13225808/s1, Table S1: Search terms used in PubMed, Scopus and ClinicalTrials.gov.
