# Supplementary material for: Uterine Preservation Treatments in Sarcomas: Oncological Problems and Reproductive Results: A Systematic Review
Source: Cancers (Basel). 2021 Nov 19;13(22):5808. doi: 10.3390/cancers13225808 (PMC8616470; doi:10.3390/cancers13225808)
Supplement: Supplementary file 1 [file cancers-13-05808-s001.zip › cancers-1458842-supplementary.pdf]

Table S1: Search terms used in PubMed, Scopus and ClinicalTrials.gov.

Database	Search term	Free-vocabulary and/or Medical Subject Headings (MeSH) terms
PubMed	**(((fertility sparing OR fertility preservation) AND (pregnancy OR Abortion Spontaneous OR assisted reproductive techniques))) AND ((Uterine Tumor Resembling Ovarian Sex Cord Tumor) OR (Uterine smooth muscle tumors uncertain malignant potential) OR sarcoma)**	((("fertiles"[All Fields] OR "fertility"[MeSH Terms] OR "fertility"[All Fields] OR "fertile"[All Fields] OR "fertilities"[All Fields]) AND ("spare"[All Fields] OR "spared"[All Fields] OR "spares"[All Fields] OR "sparing"[All Fields])) OR ("fertility preservation"[MeSH Terms] OR ("fertility"[All Fields] AND "preservation"[All Fields]) OR "fertility preservation"[All Fields])) AND ("pregnancy"[MeSH Terms] OR "pregnancy"[All Fields] OR "pregnancies"[All Fields] OR "pregnancy s"[All Fields] OR ("abortion, spontaneous"[MeSH Terms] OR ("abortion"[All Fields] AND "spontaneous"[All Fields]) OR "spontaneous abortion"[All Fields] OR ("abortion"[All Fields] AND "spontaneous"[All Fields]) OR "abortion spontaneous"[All Fields]) OR ("reproductive techniques, assisted"[MeSH Terms] OR ("reproductive"[All Fields] AND "techniques"[All Fields] AND "assisted"[All Fields]) OR "assisted reproductive techniques"[All Fields] OR ("assisted"[All Fields] AND "reproductive"[All Fields] AND "techniques"[All Fields]))) AND ((("uterine neoplasms"[MeSH Terms] OR ("uterine"[All Fields] AND "neoplasms"[All Fields]) OR "uterine neoplasms"[All Fields] OR ("uterine"[All Fields] AND "tumor"[All Fields]) OR "uterine tumor"[All Fields]) AND ("resemblance"[All Fields] OR "resemblances"[All Fields] OR "resemble"[All Fields] OR "resembled"[All Fields] OR "resembles"[All Fields] OR "resembling"[All Fields]) AND ("ovarian"[All Fields] OR "ovarians"[All Fields]) AND ("sex cord gonadal stromal tumors"[MeSH Terms] OR ("sex"[All Fields] AND "cord gonadal"[All

	Fields] AND "stromal"[All Fields] AND "tumors"[All Fields]) OR "sex cord gonadal stromal tumors"[All Fields] OR ("sex"[All Fields] AND "cord"[All Fields] AND "tumor"[All Fields]) OR "sex cord tumor"[All Fields])) OR (("uterin"[All Fields] OR "uterines"[All Fields] OR "uterus"[MeSH Terms] OR "uterus"[All Fields] OR "uterine"[All Fields]) AND ("smooth muscle tumours"[All Fields] OR "smooth muscle tumor"[MeSH Terms] OR ("smooth"[All Fields] AND "muscle"[All Fields] AND "tumor"[All Fields]) OR "smooth muscle tumor"[All Fields] OR ("smooth"[All Fields] AND "muscle"[All Fields] AND "tumors"[All Fields]) OR "smooth muscle tumors"[All Fields]) AND ("uncertainty"[MeSH Terms] OR "uncertainty"[All Fields] OR "uncertain"[All Fields]) AND ("malign"[All Fields] OR "malignance"[All Fields] OR "malignances"[All Fields] OR "malignant"[All Fields] OR "malignants"[All Fields] OR "malignities"[All Fields] OR "malignity"[All Fields] OR "malignization"[All Fields] OR "malignized"[All Fields] OR "maligns"[All Fields] OR "neoplasms"[MeSH Terms] OR "neoplasms"[All Fields] OR "malignancies"[All Fields] OR "malignancy"[All Fields]) AND ("potential"[All Fields] OR "potential s"[All Fields] OR "potentialities"[All Fields] OR "potentiality"[All Fields] OR "potentially"[All Fields] OR "potentials"[All Fields] OR "potentiate"[All Fields] OR "potentiated"[All Fields] OR "potentiates"[All Fields] OR "potentiating"[All Fields] OR "potentiation"[All Fields] OR "potentiations"[All Fields] OR "potentiative"[All Fields] OR "potentiator"[All Fields] OR "potentiators"[All Fields])) OR ("sarcoma"[MeSH Terms] OR

		"sarcoma"[All Fields] OR "sarcomas"[All Fields] OR "sarcoma s"[All Fields]))
Scopus	"Fertility sparing" AND "Uterine sarcoma" OR "Sarcoma"	(ALL FIELDS (fertility sparing) AND TITLE-ABS-KEY (uterine sarcoma OR sarcoma) AND PUBYEAR > 2011
Cochrane Central Register of Controlled Trials	"Fertility sparing" AND "Sarcoma"	Fertility sparing in Title Abstract Keyword AND sarcoma in Title Abstract Keyword - with Cochrane Library publication date Between January 2011 and June 2021 (Word variations have been searched)
